# Face image resolution and perceived attractiveness: An 8K display study

**DOI:** 10.1371/journal.pone.0348371

**Published:** 2026-06-11

**Authors:** Koji Mizukoshi, Yoshihiro Hamanaka, Xiaoguang Yang, Takaya Oishi, Hideaki Kawabata

**Affiliations:** 1 POLA Chemical Industries, Inc., Yokohama, Japan; 2 Keio University, Minato, Japan; Northumbria University, UNITED KINGDOM OF GREAT BRITAIN AND NORTHERN IRELAND

## Abstract

**Background:**

Facial attractiveness is shaped by multiple visual cues, yet the influence of image resolution on attractiveness judgments remains underexplored. Advances in display and imaging technology now allow for image presentation at resolutions approaching the limits of human spatial vision, enabling investigations under higher-fidelity spatial sampling conditions that more closely approximate retina-like detail at typical viewing distances.

**Objectives:**

This study aimed to determine whether facial image resolution, specifically matched to the spatial resolving power of the human visual system, affects perceived facial attractiveness.

**Methods:**

High-resolution facial images of 180 Japanese female models were captured using an 8K imaging system (7680 × 4320 pixels; 283.2 ppi). Low-resolution versions (35.3 ppi) were created by downsampling the originals using pixelation. Both high- and low-resolution images were presented on an 8K display in a within-subjects design. Participants rated the attractiveness of each face using a standardized 6-point scale.

**Results:**

Facial images presented at higher resolution received slightly higher attractiveness ratings than their low-resolution counterparts, as confirmed by linear mixed-effects models accounting for crossed observer and identity effects. The effect was especially pronounced for faces with higher baseline attractiveness. Age-related declines in attractiveness ratings were observed in both conditions.

**Conclusions:**

Ultra-high image resolution yields a small but reliable increase in perceived facial attractiveness, particularly for faces with higher baseline attractiveness. These findings suggest that increased spatial detail may amplify attractiveness-related cues that are otherwise diminished in conventional-resolution displays.

## 1. Introduction

Facial attractiveness plays a significant role in shaping social behaviors and interpersonal evaluations. It has been shown to influence moral judgments [1], mate selection [2], hiring decisions [3], and financial compensation assessments [4]. Given its broad impact on real-world decision-making, elucidating the mechanisms underlying the perception of facial attractiveness has become an important subject in psychological and visual science research.

In an ideal experimental setting, observers would evaluate the attractiveness of actual human faces in live interactions. However, such an approach presents considerable logistical and methodological challenges, including the need for large numbers of both participants and face models, as well as difficulties in standardizing conditions. Consequently, most studies on facial attractiveness rely on the presentation of facial images displayed on screens as a surrogate for live observation. Using this experimental setting in which facial images were presented on a screen, previous research has examined various visual features associated with facial attractiveness, such as facial symmetry [5], skin color and texture [6], and age-related changes [7]. A critical factor in such image-based studies is the fidelity with which facial features are rendered and perceived, particularly in terms of spatial sampling/detail available to the observer.

Despite this, few studies have explicitly addressed whether the resolution of the display used for image presentation matches the spatial resolution of the human eye. For example, earlier studies have typically used image resolutions in the range of 30–50 pixels per inch (ppi) [6], and even those acknowledging resolution considerations have remained limited to about 168 ppi [8]. In contrast, the spatial resolving capacity of the human retina is estimated to be approximately 300 ppi [9]. To our knowledge, no prior research has investigated facial attractiveness under visual conditions that match this retinal resolution, nor has any study systematically compared high-resolution and conventional-resolution conditions to evaluate their impact on attractiveness judgments.

Some prior studies have addressed resolution-related effects in a limited scope. Bachmann (2007) [10] found that when conventionally resolved facial images were pixelated to further reduce resolution, the perceived attractiveness of low-attractiveness faces remained unchanged, whereas the attractiveness of faces with higher baseline attractiveness decreased. Conversely, Sadr and Krowicki (2019) [11] reported that Gaussian-blurred facial images were rated as more attractive than original images. In addition, recently, Sano (2025) [12] examined how the perceived beauty or ugliness of a face can be influenced by the resolution of the face images, modulated by both down-sampling and Gaussian blurring. They found that in both cases of blurring, the more the blurring, the lower the facial beauty and the higher the ugliness are perceived. Thus, changes of the spatial resolution of facial images can have an impact on perceiving facial evaluation. However, these findings primarily concern resolution degradation below the conventional baseline (~50 ppi), leaving open the question of whether attractiveness judgments are also sensitive to resolution enhancements beyond that level.

With advances in imaging technology, particularly 8K imaging, it is now possible to create experimental conditions in which both image capture and display resolutions exceed the human eye’s spatial resolution. Such systems offer the opportunity to explore how ultra-high-resolution imagery might alter visual impression formation. While one study using 8K content reported that images of food and objects elicited enhanced impressions of realism, beauty, freshness, and taste [13], no prior research has applied this high-resolution paradigm to the evaluation of facial attractiveness.

In the present study, we employed 8K imaging and display technology to present facial images under conditions approximating the spatial resolution of the human retina. We also created lower-resolution versions of the same facial images by downsampling them to match the resolution levels used in prior studies. This dual-resolution design allowed us to investigate whether image resolution influences attractiveness judgments and to assess how these findings compare to those of previous studies that focused on lower-resolution effects.

## 2. Methods

### 2.1. Face image models and observers

Facial images of 180 Japanese female models were used as stimuli in the experiment. The age range of the models spanned from their 20s to 60s, with equal representation of each decade (36 models per group) (M = 44.5 years, SD = 13.5; range = 20–69). Forty-eight Japanese observers (24 male and 24 female) aged between their 20s and 50s participated in the study with equal representation of each decade and gender (6 participants per group) (M = 39.1 years, SD = 11.6; range = 20–58). Observers had normal or corrected-to-normal vision, and gender and age groups were equally balanced across participants.

### 2.2. Ethics statement

All tests related to this study were conducted according to the protocol approved by the ethical committee of POLA Chemical Industries, Co., Ltd., following the Declaration of Helsinki (Approval No. 2021-F-013; March 11, 2021, Approval No. 2021-F-073; October 18, 2021). Written informed consent was received from all participants and face models to publish these case details. Participants were prospectively recruited via open public calls from March 24, 2021, to May 31 2021 for 2021-F-013 and October 23, 2021, to December 24 2021 for 2021-F-073. The face image shown in the schematic illustration in Fig 2 is a non-identifiable composite average face used solely for illustrative purposes to explain the experimental procedure. It does not depict any identifiable individual. The actual experimental stimuli consisted of face photographs of real individuals, as described in the Methods section.

### 2.3. Image acquisition and processing

Facial images were captured using an 8K (i.e., 8192 x 4320) digital cinema camera (Monstro, Red Digital Cinema Camera Company, USA) equipped with a 65-mm fixed focal length lens (Carl Zeiss AG, Germany). The camera was positioned 140 cm from the model, with the lens aligned at eye level. Six Astra 6X Bi-Color LED lights (Litepanels, United Kingdom) were arranged asymmetrically around the model to simulate a typical office lighting environment. A horizontal reflector was placed at abdominal height to provide upward fill lighting.

**Fig 1:**
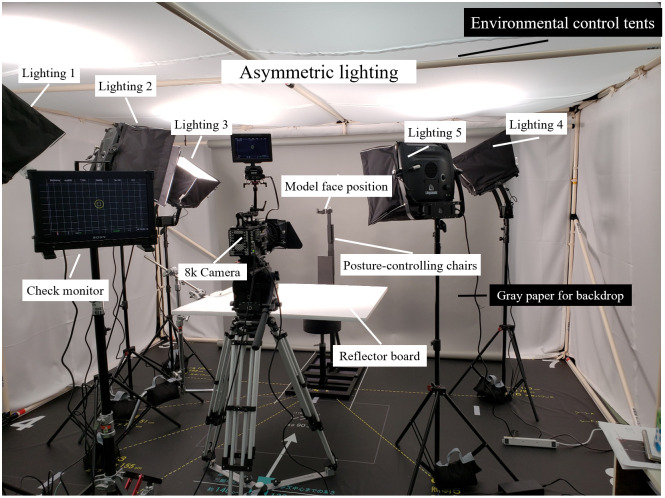
Photography environment for capturing face images.

All image acquisition was conducted inside a white-curtain-enclosed tent to maintain constant lighting and environmental conditions (Fig 1). During shooting, exposure and focus were adjusted to optimize image clarity. Post-capture, the images were adjusted for color temperature, brightness, and saturation using visual matching against the live model as seen on a 32-inch 8K display (8M-B32C1, SHARP CORPORATION, Japan) at a viewing distance of 1 meter. A graphics specialist (Amana Images, Japan) supported the image processing and quality adjustments.

Facial images were standardized with the following conditions: the Frankfurt horizontal plane was aligned, models kept a neutral facial expression with eyes directed toward the lens, mouth closed, and head upright. The ears were digitally removed along the facial outline. The final image was cropped into a square region centered on the centroid of the triangle formed by the inner canthi and the midpoint of the upper lip. This square included the top of the forehead and the bottom of the chin. The background was set to a mid-gray (RGB: 128, 128, 128).

To control for visual angle, the width of each face image was adjusted to subtend 8° of visual angle at a viewing distance of 100 cm, corresponding to an average real-world face width of 14 cm, or 1,562 pixels on the display. The face images were centered on screen. These images were designated as the high-resolution condition, corresponding to 283.2 pixels per inch (ppi), or 111.5 px/cm. Low-resolution images were created by downsampling to be pixelizing the high-resolution images to one-eighth their original resolution (35.3 ppi, 13.9 px/cm). All image processing was performed using Adobe Photoshop 23 (Adobe Systems, CA, USA).

### 2.4. Attractiveness rating task

In the attractiveness rating task, The experiment was conducted in a darkened room to minimize environmental distractions and maintain consistent viewing conditions. To simulate the typical distance during face-to-face interpersonal communication, observers sat 100 cm from the display, with the center of the screen aligned to their eye level.

**Fig 2:**
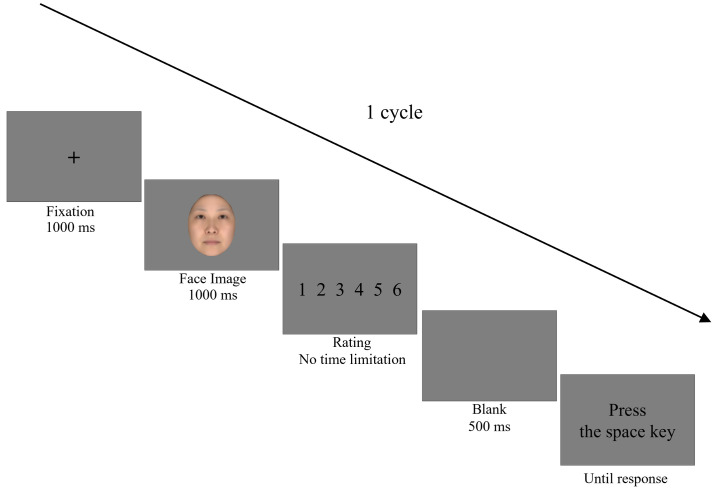
Evaluation of attractiveness from face images. The face shown is a non-identifiable composite average face.

Each trial began with the presentation of a fixation cross for 1,000 ms, followed by the facial image (either high- or low-resolution) for 1,000 ms. Observers rated the attractiveness of each face using a 6-point Likert scale (1 = not attractive, 6 = highly attractive). There was no time limit for rating. After each response, a 500-ms blank screen was presented. Observers then initiated the next trial by pressing the spacebar (Fig 2).

Each observer viewed a total of 360 trials—180 images per resolution condition. Image presentation was randomized across trials, with model age group and resolution condition counterbalanced. Stimulus presentation and response collection were implemented using E-Prime 3.0 software (Psychology Software Tools, Inc., USA).

### 2.5. Statistical analysis

Statistical analyses were conducted in MATLAB (R2025b; MathWorks). To account for the crossed repeated-measures structure (multiple ratings nested within observers and face identities), attractiveness ratings were analysed using linear mixed-effects models (LMMs; fitlme). The dependent variable was the 6-point attractiveness rating. Fixed effects included image resolution (High vs. Low), model age period (20s–60s), and their interaction (Resolution × AgePeriod). Random intercepts and random slopes for Resolution were specified for both observers and face identities: (1 + Resolution | Subject_No) + (1 + Resolution | Model_faceID).

To evaluate the Resolution × AgePeriod interaction, we compared the full model with a reduced model without the interaction (Resolution + AgePeriod) using a likelihood-ratio test (LRT); both models were fitted with maximum likelihood (ML). Final parameter estimates were obtained using restricted maximum likelihood (REML), and fixed effects were evaluated with Satterthwaite-approximated degrees of freedom.

We additionally report marginal and conditional R² for the LMMs (variance explained by fixed effects only vs. the full model including random effects), following Nakagawa and Schielzeth (2013) [14].

For descriptive purposes and comparability with prior work, we also computed Spearman’s rank correlations between model age and mean attractiveness, computed separately for each resolution condition.

To test whether the effect of resolution varied with baseline attractiveness, we conducted additional LMM moderation analyses. Baseline attractiveness was defined for each face identity as the mean rating averaged across both resolution conditions and all observers. We tested (i) a continuous moderator model including the Resolution × baseline-attractiveness interaction and (ii) a median-split model comparing faces with higher vs. lower baseline attractiveness. Interaction terms were evaluated using ML-based LRTs, and final parameter estimates were obtained using REML.

## 3. Results

High-resolution facial images of 180 Japanese female models were captured using the 8K imaging system described above, and each image was downsampled to produce a corresponding low-resolution version.

**Fig 3:**
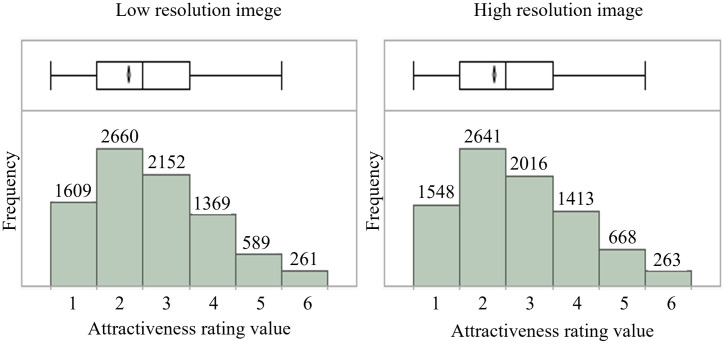
Histogram of 8640 evaluation results for attractiveness ratings across different image resolutions.

A total of 48 participants rated the attractiveness of 360 facial images (180 identities × 2 resolutions) on a 6-point scale, yielding 17,280 observations in total (8,640 per resolution). Across both resolution conditions, all rating levels from 1 to 6 were used, with ratings of “2” being most frequent (Fig 3).

To assess the effect of image resolution while accounting for the crossed repeated-measures structure of the data (multiple ratings nested within observers and face identities), we analysed trial-level ratings using linear mixed-effects models. The model included Resolution (High vs. Low), AgePeriod (20s–60s), and their interaction as fixed effects, with random intercepts and random slopes for Resolution for both observers and face identities.

The Resolution × AgePeriod interaction was not significant (ML-based LRT: χ²(4) = 2.94, p = 0.567; see also F(4, 2009.1) = 0.736, p = 0.568), indicating that the resolution effect did not reliably differ across age periods. There was, however, a small but reliable main effect of Resolution, F(1, 49.23) = 6.93, p = 0.011, such that high-resolution images were rated slightly more attractive than low-resolution images. This small effect is visualized descriptively in Fig 5. In terms of variance explained, the fixed effects accounted for a modest proportion of variability (marginal R² = 0.130), whereas the full model including random effects captured substantially more variance (conditional R² = 0.574).

**Fig 4:**
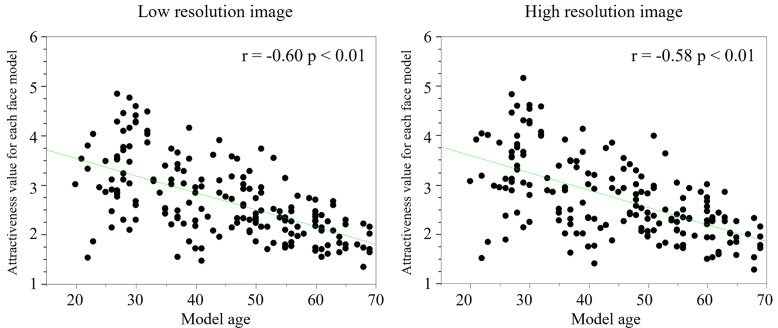
Relation between age and average attractiveness rating for face images of different resolutions.

AgePeriod showed a robust main effect, F(4, 175) = 24.30, p = 5.28 × 10 ⁻ ¹⁶, with higher ratings for faces in their 20s–30s and lower ratings for faces in their 50s–60s. For descriptive purposes, we also examined the relationship between model age and identity-level mean attractiveness separately for each resolution condition. In both conditions, attractiveness decreased with age (low resolution: r = −0.60, p < 0.001; high resolution: r = −0.58, p < 0.001; Fig 4).

### 3.1. Moderation by baseline attractiveness (LMM-based)

We next tested whether the effect of resolution depends on baseline attractiveness of the face identity. Baseline attractiveness was defined for each identity as its mean rating averaged across both resolutions and all observers. In a continuous moderation model, the Resolution × baseline-attractiveness interaction was significant (ML-based LRT: χ²(1) = 7.14, p = 0.00753), indicating that the resolution benefit increases with baseline attractiveness. This pattern is visualized descriptively in Fig 6A.

For comparability with the original median-split approach, we also contrasted faces with higher vs. lower baseline attractiveness. The Resolution × group interaction was significant (ML-based LRT: χ²(1) = 8.92, p = 0.00281), consistent with a pronounced resolution benefit in the higher-baseline-attractiveness group and little to no benefit in the lower-baseline-attractiveness group (Figs 6B and 7). Throughout, Δ denotes the difference High − Low.

## 4. Discussion

In this study, we employed 8K imaging technology to capture and present facial images of Japanese female models at a spatial resolution (283.2 ppi) approaching that of the human retina. To simulate realistic everyday environments, we used asymmetrical lighting, reflecting the natural uneven distribution of light in real-world conditions. This setting allowed us to investigate facial attractiveness not only in terms of facial coloration but also three-dimensional facial features that are likely to influence impression formation. The construction of an experimental environment tailored to human eye resolution (≈8K) in such everyday communication scenarios represents a significant departure from previous research. Low-resolution images (35.3 ppi) were created from high-resolution originals using pixelation, a common downsampling method that retains resolution-specific structure without introducing artificial blur, unlike Gaussian filtering. These high- and low-resolution images were evaluated by participants for facial attractiveness to examine the effect of resolution.

**Fig 5:**
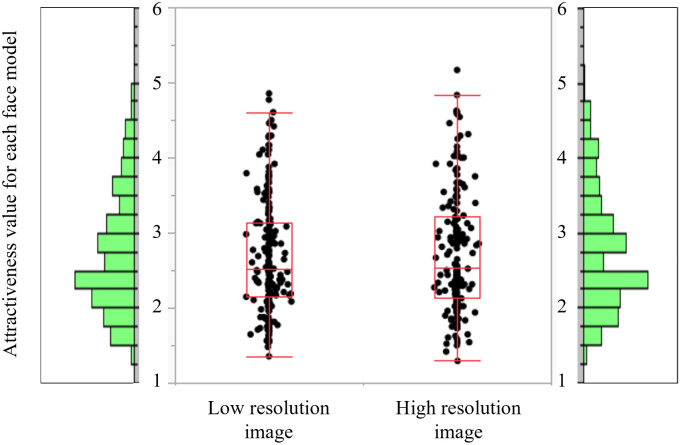
Comparison of attractiveness ratings between high- and low-resolution face images. Points show identity-level mean attractiveness ratings (averaged across observers) for each resolution condition, with boxplots and marginal distributions. The resolution effect was evaluated using a linear mixed-effects model accounting for crossed observer and identity effects (random intercepts and random slopes for Resolution): F(1, 49.23) = 6.93, p = 0.011.

**Fig 6:**
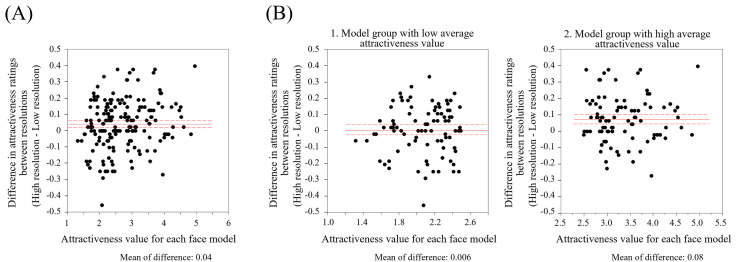
Baseline-attractiveness moderation of the resolution effect (High – Low) on attractiveness ratings. **(A)** Relationship between each identity’s baseline attractiveness (identity-level mean rating averaged across both resolutions and all observers) and the identity-level resolution difference (High − Low). **(B)** The same resolution difference shown separately for faces with higher vs. lower baseline attractiveness (median split). Moderation was tested with LMMs: the continuous interaction (Resolution × baseline attractiveness) was significant (ML-LRT χ²(1) = 7.14, p = 0.00753), and the median-split interaction (Resolution × group) was also significant (ML-LRT χ²(1) = 8.92, p = 0.00281).

**Fig 7:**
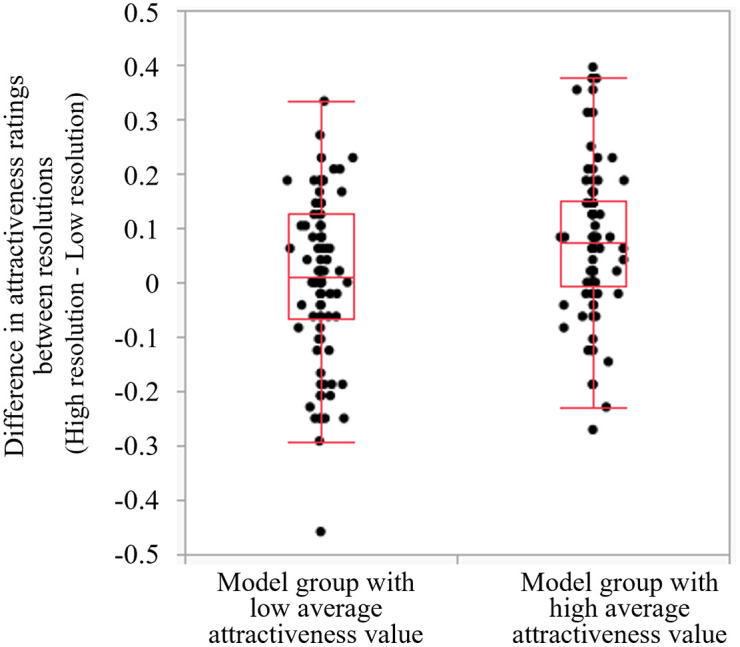
Resolution effect (High – Low) by baseline-attractiveness group. Distributions of identity-level resolution differences (High − Low) for faces with higher vs. lower baseline attractiveness (median split). Group differences in the resolution effect were evaluated using an LMM with the Resolution × group interaction: ML-LRT χ²(1) = 8.92, p = 0.00281.

Consistent with prior findings [7], attractiveness ratings decreased with increasing model age across both resolutions. Moreover, high-resolution images were rated slightly more attractive on average than their low-resolution counterparts (Fig 5). Importantly, the resolution effect did not show evidence of varying systematically across age periods. In addition, consistent with our LMM moderation analyses, the resolution advantage was larger for faces with higher baseline attractiveness (Figs 6 and 7). Observer feedback also suggested that 8K displays offered a greater sense of depth and realism in facial representation. This anecdotal impression was echoed by the experimenters themselves, who informally reported that the high-resolution images appeared notably more three-dimensional and lifelike during testing. These findings align with previous studies on 8K image presentation, where 8K conditions enhanced impressions of depth, realism, and presence for a range of visual stimuli including people, animals, and landscapes [15]. Additional effects have been reported on both perceptual elements (e.g., vividness, contrast, color expression) and cognitive evaluations (e.g., realism, beauty, temperature), particularly those involving top-down processing [13]. Our findings suggest that such enhanced image features were present in the high-resolution face images, thereby contributing to increased attractiveness ratings, especially for faces with higher baseline attractiveness.

Here, baseline attractiveness refers to the identity-level mean rating averaged across both resolution conditions and all observers. Relatedly, cosmetic studies have shown that contrast modifications—such as those introduced by makeup—can significantly affect perceived attractiveness [16,17]. These studies have also proposed dual mechanisms involving immediate, reflexive impressions and more deliberate, conscious evaluations [16]. We speculate that high-resolution imaging may similarly trigger both types of processing, particularly in images of highly attractive faces.

Interestingly, Bachmann (2007) [10] demonstrated that pixelating conventionally-resolved images could sometimes enhance the attractiveness of faces with higher baseline attractiveness. Although that study investigated the effect of resolution reduction, and our study addressed resolution enhancement, both point to a shared pattern: attractiveness judgments can be modulated by changes in image resolution, especially for faces with higher baseline attractiveness. The pixel density in Bachmann’s study was estimated to be in the tens of ppi range, though exact rendering conditions were not provided.

Given the established role of physical symmetry [18] and averageness [19] in facial attractiveness, future research should explore which specific image features in 8K resolution are driving increased ratings. A promising approach would be to employ deep learning to build predictive models. By labeling face identities according to whether attractiveness increases under high-resolution presentation (i.e., a positive High–Low difference) or shows little to no increase, we may be able to extract informative image features from neural networks.

Because the present study contrasts two rendering conditions (retina-like high resolution vs. a pixelated low-resolution version), the observed difference should be interpreted as a relative preference for the high-resolution rendering under our display conditions, rather than definitive evidence that ultra-high resolution per se “enhances” attractiveness. Without intermediate resolution levels or a non-pixelated low-resolution baseline, we cannot disentangle an increase driven by higher spatial detail from a decrease driven by low-resolution degradation. Moreover, pixelation introduces blocky artifacts; although chosen to avoid blur confounds, it may not fully reflect how lower resolution is naturally encountered. Future work should therefore include multiple intermediate resolution levels and alternative degradation methods (e.g., bicubic downsampling, optical blur, compression) to strengthen causal interpretability and ecological validity.

More broadly, our stimuli were static, standardized 2D images presented on a flat display, and thus do not capture key components of real-world face perception such as dynamic facial motion, three-dimensional structure, gaze contingencies, and interactive social context. Therefore, the present paradigm should be interpreted as isolating the contribution of spatial sampling/detail under controlled conditions, rather than providing a comprehensive test of ecological validity. This distinction is critical when considering whether ultra-high-resolution (8K) imaging is necessary for research on face perception, especially given the modest effect sizes and substantial equipment costs. Our findings suggest that, while the human visual system may not require such high spatial resolution for basic attractiveness judgments, subtle yet reliable increases in ratings indicate that fine-grained spatial details modulate the perception of facial aesthetics. This is consistent with previous evidence indicating that lower-resolution cues are often sufficient for certain social judgments. However, our results contrast with those of Holzleitner et al. (2021), who found that 3D facial images did not provide more accurate judgments of physical characteristics than 2D images. The present study suggests that increased image fidelity may amplify certain visual cues for subjective aesthetic evaluations. Thus, 8K technology is a high-precision tool for isolating specific visual mechanisms, even if its practical advantage in everyday attractiveness assessment is limited. Furthermore, our results simultaneously suggest the importance of considering additional facial information, such as enhanced makeup or skin condition, which can increase attractiveness even at low resolutions, just as it does under high-resolution conditions.

Another limitation is that we did not include non-face control categories. Therefore, we cannot determine whether the observed resolution-related difference is specific to facial attractiveness mechanisms or reflects a broader image-quality effect (e.g., higher realism or general aesthetic appeal) that could apply to many object categories. Future work should include non-face comparison stimuli (e.g., objects or landscapes) and/or additional judgments (e.g., realism and general liking) to dissociate face-specific contributions from general image-quality influences.

Our results also suggest that different resolution levels emphasize different facial features, both of which may positively influence attractiveness under certain conditions. However, the number of faces receiving high attractiveness ratings was limited (Fig 3), and future studies should employ facial databases with a more balanced or normally distributed range of attractiveness scores. Therefore, the “higher baseline attractiveness” group obtained via a median split should be interpreted as relatively higher *within this sample* rather than as a truly high-attractiveness subset. Consequently, the moderation effect should be generalized cautiously and warrants replication using stimulus sets spanning a wider range of attractiveness. Additionally, poor skin condition or injuries may be more noticeable at higher resolutions, which could reduce attractiveness. This suggests that higher resolution may influence the baseline attractiveness of the subject. Because baseline attractiveness in the present analyses was derived from the same rating data, future work should replicate the moderation effect using an independent baseline measure (e.g., separate observers or pre-ratings). While our sample included a wide range of model ages, all models were evaluated using the same rating scale, which resulted in lower average attractiveness ratings and smaller variance among older models (Fig 4). Future designs might benefit from normalization or stratification techniques to control for such age-related differences in perceived attractiveness. In the present study, we prioritized evaluating all images within the same specialized 8K experimental environment to maintain internal validity. Since this study examines how high-fidelity spatial sampling that matches the limits of human vision influences judgments of attractiveness, it was crucial to ensure consistent display and viewing conditions across all ratings before generalizing the results to external, standardized databases.

High-resolution imaging offers a novel lens through which to investigate subtle visual features of human skin and facial structure. The present results suggest that such fine-grained visual information can contribute to impression formation, with the impact being most pronounced for faces with higher baseline attractiveness. Insights derived from such investigations could inform cosmetic innovations and improve realism in virtual environments such as head-mounted displays and virtual try-on technologies.

## 5. Conclusion

This study evaluated facial attractiveness using images with a spatial resolution close to that of the human retina. A small but reliable difference in attractiveness ratings was observed between the high-resolution images and those with lower resolutions typically used in previous studies, as confirmed by linear mixed-effects models accounting for crossed observer and identity effects. Moreover, the increase in attractiveness ratings due to high resolution was larger for faces with higher baseline attractiveness. These findings suggest that specific visual features contributing to attractiveness may become more salient at higher resolutions, and that such features selectively enhance the perceived attractiveness of faces with higher baseline attractiveness.
